# Scalable Isolation of Human Umbilical Cord MSC-Derived Exosomes and Their Therapeutic Potential in Osteoarthritis

**DOI:** 10.3390/bioengineering13070770

**Published:** 2026-06-30

**Authors:** Chao Zhou, Shimei Wu, Yanyi Zeng, Xueyan Liu, Shiye Wu, Ke Chen, Junrong Wu, Haibin Yin, Yuanyuan Zhou

**Affiliations:** 1 School of Pharmaceutical Sciences (Shenzhen), Shenzhen Campus of Sun Yat-Sen University, 66 Gongchanglu Road, Guangming District, Shenzhen 518107, China; zhouch79@mail2.sysu.edu.cn; 2 Guangzhou National Laboratory, Guangzhou International Bio Island, No. 9 Xingdaohuanbei Road, Guangzhou 510005, China; 3 Guangzhou Anjie Biomedical Technology Co., Ltd., Guangzhou 510005, China; wushim439@163.com (S.W.); liuxy@anjiebiomed.com (X.L.); wujr@anjiebiomed.com (J.W.); 4 School of Pharmaceutical Sciences, Guangzhou University of Chinese Medicine, Guangzhou 510006, China; 13660831517@163.com; 5 School of Animal Science and Technology, Foshan University, Foshan 528225, China; 18998449810@163.com (S.W.); 19074908377@163.com (K.C.); 6 School of Agriculture and Bioengineering, Foshan University, Foshan 528225, China

**Keywords:** osteoarthritis, human umbilical cord MSCs, exosomes, cartilage repair, inflammation

## Abstract

Osteoarthritis (OA) is a prevalent degenerative joint disorder characterized by cartilage degradation, synovial inflammation, and osteophyte formation, yet effective therapies that alter disease progression remain absent. Mesenchymal stem cell-derived exosomes (MSC-EXOs), as a cell-free regenerative medicine strategy, have shown great potential in the treatment of osteoarthritis. In this study, we successfully isolated and purified exosomes derived from human umbilical cord mesenchymal stem cells using a scalable tangential flow filtration (TFF)–chromatography platform and evaluated their therapeutic effects on OA model induced by anterior cruciate ligament transection (ACLT). OARSI scores were significantly reduced compared with the ACLT group (*p* < 0.01). Exosomes administration markedly reduced osteophyte formation, preserved cartilage structure, enhanced collagen II expression (*p* < 0.01), and suppressed MMP13-mediated matrix degradation (*p* < 0.05) compared with the ACLT group. The treatment also significantly decreased pro-inflammatory cytokines, indicating alleviation of the inflammatory microenvironment. Transcriptomic profiling further revealed genes and pathways potentially associated with exosome treatment. These findings suggest that hUC-MSC-EXOs isolated and purified using TFF–chromatography exert robust chondroprotective and immunomodulatory effects, supporting their potential as an effective cell-free therapeutic candidate for OA.

## 1. Introduction

Osteoarthritis (OA) is a highly prevalent degenerative joint disease, and its growing incidence has placed a significant burden on public health systems. The global burden of OA continues to increase owing to population aging, obesity, and longer life expectancy, affecting more than 500 million people worldwide and imposing substantial healthcare and socioeconomic burdens [1,2]. Pathologically, osteoarthritis is now recognized as a whole-joint disease characterized by progressive articular cartilage degradation, subchondral bone remodeling, synovial inflammation, and osteophyte formation. These pathological changes interact to promote chronic joint degeneration and functional impairment. Despite advances in symptomatic management, effective strategies capable of restoring cartilage homeostasis and preventing disease progression remain limited [3,4,5,6].

Current clinical treatments for osteoarthritis (OA) remain markedly limited: joint immobilization used in the early to middle stages offers only short-term symptom relief and fails to reverse cartilage damage; intra-articular injections of hyaluronic acid, corticosteroids, and other supplementary agents may temporarily improve joint function, yet they are insufficient to stimulate cartilage regeneration and show poor long-term efficacy [7]. In recent years, stem cell-based therapy has emerged as a research hotspot for repairing damaged tissues. Mesenchymal stem cells (MSCs) secrete growth factors and cytokines with trophic, chemotactic, and immunosuppressive properties, and animal studies have demonstrated their potential in regenerating musculoskeletal tissues [8,9]. However, direct transplantation of stem cells still poses several limitations, such as immune rejection, tumorigenicity, and risks of chromosomal instability [10]. Therefore, developing alternative strategies that can harness the beneficial properties of stem cells while avoiding these safety concerns has become a central focus in OA-related therapeutic research.

Exosomes, extracellular vesicles with a diameter of approximately 30–150 nm, are actively secreted by cells and carry enzymes, growth factors, and both coding and non-coding RNAs derived from their parental cells. They mediate intercellular communication by delivering bioactive molecules to recipient cells, thereby regulating diverse biological functions [11,12,13]. Recent studies have demonstrated that stem cell-derived exosomes not only retain the core regenerative capacities of their parental stem cells but also circumvent the safety risks associated with stem cell-based therapy, showing great promise in the treatment of degenerative diseases [14,15]. Multiple studies have reported that mesenchymal stem cell-derived exosomes exert notable therapeutic effects on OA. Exosomes derived from human umbilical cord mesenchymal stem cells (hUC-MSCs) can attenuate articular cartilage degeneration, markedly suppress osteophyte formation, and reduce the production of pro-inflammatory cytokines such as IL-1β in the knee joint, thereby alleviating inflammation and slowing the progression of knee OA [16]. Exosomes derived from bone marrow mesenchymal stem cells (BMSCs) have been shown to attenuate IL-1β-induced inflammatory responses and promote chondrocyte survival and tissue repair, effectively promote cartilage repair and extracellular matrix synthesis in OA rats, and reduce knee joint pain; moreover, they can act on macrophages to promote M2 polarization and enhance the secretion of anti-inflammatory cytokines [17]. Synovial mesenchymal stem cells (SMSCs) possess stronger chondrogenic stimulatory capacity than BMSCs, and exosomes derived from SMSCs promote chondrocyte proliferation and migration [18]. Exosomes from adipose-derived mesenchymal stem cells (AMSCs) facilitate cartilage repair by promoting the infiltration of regenerative M2 macrophages into defect sites [19]. Collectively, these findings highlight the therapeutic potential of MSC-derived exosomes in OA treatment [20,21].

Among exosomes derived from various stem cell types, human umbilical cord mesenchymal stem cell-derived exosomes (hUC-MSC-EXOs) exhibit distinct advantages. The mRNAs carried by stem cell-derived exosomes can influence chondrocyte function through modulating histone modifications and DNA methylation, while their miRNA cargo can target and suppress key OA-related pathogenic factors such as RUNX2, MMP-13, and ADAMTS-5, thereby maintaining the balance between anabolic and catabolic activities in cartilage [18,22,23]. However, the therapeutic efficacy of stem cell-derived exosomes is constrained by multiple factors. Although stem cells from different tissues share similar phenotypes, the composition of their exosomes can vary substantially due to differences in parental cell origin, culture conditions, and other environmental factors, resulting in distinct profiles of proteins, nucleic acids, and cytokines. Consequently, exosomes derived from different stem cell sources exert heterogeneous effects on recipient cells [24,25,26]. Among various stem cell types, human umbilical cord-derived mesenchymal stem cells (hUC-MSCs) offer several advantages, including ease of isolation and expansion, cost-effectiveness, non-invasive collection procedures, and low risk of infection. They have gained significant attention due to their strong proliferative capacity, broad differentiation potential, and low immunogenicity [27]. Compared with MSCs derived from bone marrow or adipose tissue, hUC-MSC-derived exosomes carry a richer repertoire of anti-inflammatory and pro-regenerative molecules, enabling their use in treating a range of inflammation-related disorders. These exosomes can also exert stable reparative effects under relatively low immunogenic conditions, making them one of the most promising sources of extracellular vesicles for therapeutic applications [28]. Although MSC-derived extracellular vesicles have emerged as promising cell-free therapeutics with advantages such as low immunogenicity, and reduced tumorigenic risk compared with cell-based therapies, several challenges remain. Variability in donor characteristics, isolation procedures, and vesicle composition may influence therapeutic consistency and efficacy. In addition, emerging evidence suggests that extracellular vesicle-mediated intercellular communication may exert context-dependent biological effects, including the modulation of cellular senescence and tissue homeostasis, highlighting the need for further mechanistic and translational studies [29].

At present, research on the application of hUC-MSC-derived exosomes to treat osteoarthritis remains in its early stages, and their in vivo reparative mechanisms, molecular targets, and regulatory networks are still unclear. Existing evidence suggests that MSC-derived exosomes may modulate chondrocyte metabolic balance and synovial inflammation through signaling pathways such as PI3K/Akt, NF-κB, and TGF-β/Smad. They significantly reduce the levels of TNF-α, IL-1β, and IL-6, which are key inflammatory mediators that contribute to cartilage destruction and joint inflammation [30]. They can also enhance endothelial cell activity and angiogenesis to promote osteogenesis and accelerate fracture healing, restore stem cell potential, and mitigate local bone loss associated with age-related osteoporosis [31]. However, the precise molecular events by which they repair cartilage and delay OA progression remain to be elucidated. This lack of mechanistic understanding, together with the insufficient preclinical studies, continues to hinder the development of targeted therapeutic strategies.

Furthermore, the clinical application of hUC-MSC-EXOs faces the challenge of large-scale production. Current mainstream isolation techniques, such as density gradient centrifugation and ultracentrifugation, have inherent limitations including being time-consuming, small scale, low yield, and requiring expensive equipment [32,,33,,34,35,36]. The mechanical forces generated during these processes may also disrupt the membrane structure and function of exosomes [34,35]. Additionally, the lack of standardized manufacturing protocols leads to batch-to-batch variability, directly affecting the stability of therapeutic efficacy [37]. Consequently, existing isolation techniques are insufficient to meet the requirements for large-scale exosome preparation. Establishing an efficient, rapid, and scalable isolation method is crucial for the development of MSC-EXOs-based therapeutic strategies.

Based on these considerations, this study focused on the scalable isolation method of exosomes derived from human umbilical cord-derived mesenchymal stem cells and their therapeutic effects and mechanisms on osteoarthritis. We first isolated MSC-EXOs using tangential flow filtration (TFF) and chromatography purification, and then characterized the particle size, concentration, morphology, and marker protein expression of the exosomes using nanoparticle tracking analysis (NTA), transmission electron microscopy, and Western blotting, respectively. Subsequently, a rat OA model was established via ACLT surgery to evaluate the therapeutic effects of hUC-MSC-derived exosomes. Transcriptome sequencing was further performed to identify potential molecular targets and regulatory pathways involved in their therapeutic action. This study aims to provide experimental evidence for the preclinical development of stem cell-derived exosome-based therapeutics for OA and to support the standardized development and translational application of exosome products in OA treatment.

## 2. Materials and Methods

### 2.1. Culture and Characterization of hUC-MSCs-EXO

hUC-MSCs were provided by Guangzhou Anjie Biomedical Technology Co., Ltd. (Guangzhou, China). The cells were cultured in serum-free medium at 37 °C in a humidified incubator containing 5% CO_2_. Upon reaching 80–90% confluence, they were digested with diluted recombinant trypsin solution (CTS™ TrypLE™ Select Enzyme, Gibco, Waltham, MA, USA) and subsequently subcultured. The culture supernatant of passage 5 (P5) cells cultured for 48–72 h was collected for the isolation and purification of exosomes. Flow cytometry was performed to detect the expression of specific surface markers on UC-MSCs, including CD73, CD90, and CD105, as well as CD19, CD34, CD45, CD11b, and HLA-DR.

### 2.2. Isolation and Purification of hUC-MSCs

**Figure 1:**
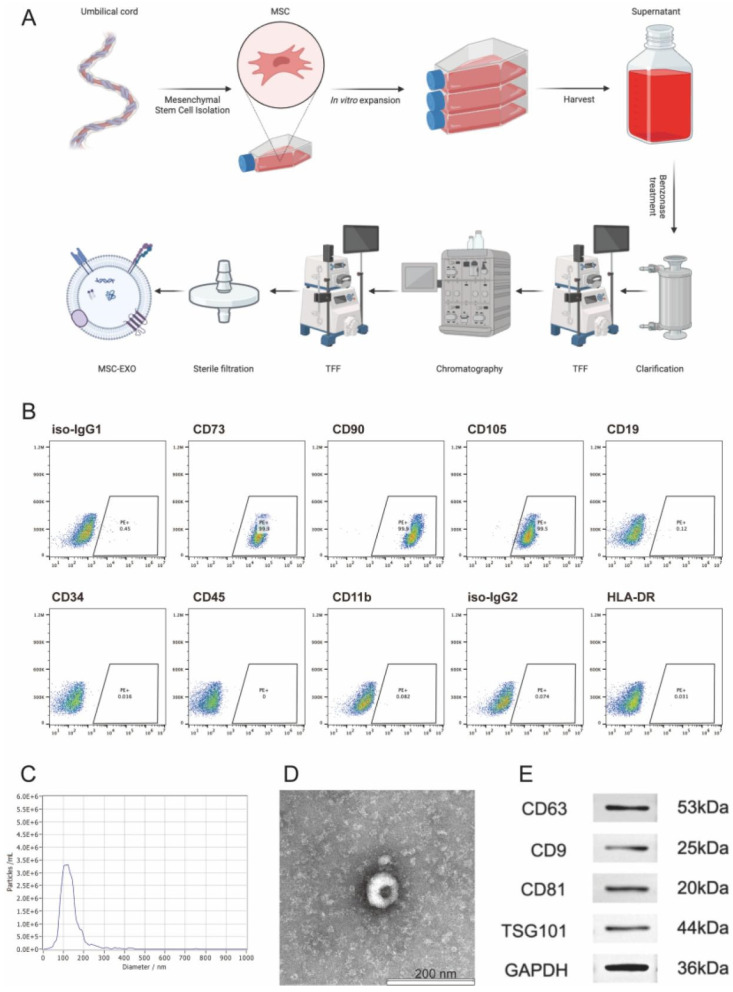
Purification and identification of hUC-MSC-EXOs. (**A**) Scheme of exosomes isolation and purification by tangential flow filtration (TFF) and Capto Core 400 multimodal chromatography (figures were created with BioRender.com). (**B**) Flow cytometry analysis showed that hUC-MSCs expressed specific markers on their surface. (**C**) Particle size distribution of hUC-MSCs-EXOs analyzed by nanoparticle tracking analysis (NTA). (**D**) Representative transmission electron microscopy images of exosomes. (**E**) Representative Western blot images showing EV-associated marker proteins CD63, CD9, CD81, and TSG101.

hUC-MSC-EXOs were isolated and purified using a simple, efficient, and scalable method, as illustrated in Figure 1A. Briefly, Benzonase (Vazyme Biotech, Nanjing, China) at a final concentration of 1 IU/mL and MgCl_2_ at a final concentration of 2 mM were added to the collected UC-MSC culture supernatant, mixed thoroughly, and incubated overnight at 4–8 °C. The supernatant was then clarified using a depth filter (Sartopure^®^ GF Plus Midicaps^®^ 0.65 µm Size 7, Sartorius, Göttingen, Germany) to clear cells and cell debris. The clarified supernatant was concentrated 8- to 10-fold by tangential flow filtration (TFF) using the KrosFlo^®^ KR2i TFF System (Repligen, Waltham, MA, USA) equipped with 300 kDa MWCO Spectrum hollow fibers (Repligen). The transmembrane pressure (TMP) was maintained at 2.0–3.0 psi. Subsequently, the concentrated sample was subjected to chromatography purification on an NGC 100 Medium-Pressure Chromatography System (Bio-Rad, Hercules, CA, USA) with a chromatographic column packed with Capto Core 400 resin (Cytiva, Marlborough, MA, USA), and the flow-through fraction was collected. The collected exosomes were further concentrated 8- to 10-fold using 100 kDa MWCO Spectrum hollow fibers (Repligen) via TFF, and the buffer was exchanged to 0.9% saline. Finally, the product was sterilized by filtration through a 0.22 μm filter, aliquoted, and stored at −80 °C for subsequent experiments.

### 2.3. NTA of Exosome Particle Concentration and Size Distribution

The sample chamber was rinsed with deionized water, and the instrument was calibrated using 100 nm polystyrene bead standards diluted at 1:250,000. After rinsing the chamber again with 1× PBS buffer, the samples were diluted 1000-fold in 1× PBS and subjected to nanoparticle tracking analysis (NTA) using a ZetaView PMX-120 system (Particle Metrix, Inning am Ammersee, Germany) to determine exosome particle concentration and size distribution.

### 2.4. Transmission Electron Microscopy (TEM) for Morphological Identification of Exosomes

Parafilm was placed flat on a glass slide, and a copper grid was positioned on the Parafilm using tweezers. A 10-μL aliquot of exosome sample was pipetted onto the grid and incubated for 10 min, after which excess liquid was wicked away with filter paper. Subsequently, 10 μL of 2% uranyl acetate was added for negative staining for 1–3 min, followed by removal of residual stain with filter paper. The grid was air-dried at room temperature for 10 min and then imaged under a transmission electron microscope at 100 kV.

### 2.5. Western Blot Analysis of Exosomal Marker Proteins

Western blot analysis was used for qualitative characterization of EV markers. Western blot images are presented as representative results for qualitative characterization. Exosomal proteins were extracted using RIPA lysis buffer at threefold the sample volume, and protein concentrations were quantified using a BCA assay kit. Sample loading buffer was added at a 4:1 ratio, followed by denaturation at 95 °C for 5 min. Proteins were separated by SDS-PAGE (8.0–12.5%), transferred onto nitrocellulose membranes, and blocked with 5% bovine serum albumin for 1 h to prevent nonspecific binding. Target bands were excised and incubated overnight at 4 °C with specific primary antibodies. After three washes with Tris-buffered saline containing 0.1% Tween-20 (TBST, 15 min each), membranes were incubated with HRP-conjugated secondary antibodies at room temperature for 1 h, washed again with TBST, and then treated with freshly prepared ECL substrate. Chemiluminescent signals were detected and analyzed for band optical density using ImageJ software (1.53 versio).

### 2.6. Animals

All experimental procedures were carried out in accordance with the guidelines in “the Animal Management Regulations” of the Institutional Animal Care and Use (approval code: KSSW 2025-7-25-0A, KSSW 2025-7-25-0C). Thirty-six SD (male, 8 weeks old) rats were purchased from Charles River Laboratories. A total of 12 animals were randomly assigned to each group. We established a rat OA model via transection of the anterior cruciate ligaments (ACLT). Rats were anesthetized by intraperitoneal injection of pentobarbital sodium (40 mg/kg) prior to surgery. After 2 weeks post-surgery, a total volume of 100 μL saline or hUCB-MSC-EXO was injected into the knee joint cavity with a microinjector. At the end of the experiment, animals were euthanized by carbon dioxide inhalation using a gradual-fill method. Death was confirmed by cessation of respiration and cardiac activity.

### 2.7. Micro-CT Analysis

After treatment, rat knee joints were washed with pre-chilled saline and preserved in alcohol. The samples were placed into a micro-computed tomography (micro-CT) scanning chamber, and the subchondral bone was selected as the region of interest (ROI). After setting the scanning parameters, three-dimensional reconstruction was performed to analyze cartilage-related morphological parameters.

### 2.8. Histological Staining

For histological staining, knee joint sections were cut at a thickness of 4 μm along the sagittal plane. The degree of destruction of the knee cartilage was evaluated using Safranin O/Fast Green staining (Leagene, Beijing, China) while the Osteoarthritis Research Society International (OARSI) OA histopathology assessment system was applied to assess the severity of cartilage degeneration. Synovitis score was determined by hematoxylin and eosin staining (H&E, Leagene, China).

### 2.9. Immunohistochemical Staining

Prepared tissue sections were incubated in 3% hydrogen peroxide for 10 min to eliminate endogenous peroxidase activity. After serum blocking, primary antibodies (Col II or MMP-13) were applied and incubated overnight at 4 °C. Sections were then incubated with HRP-conjugated secondary antibodies at room temperature for 1 h, followed by detection using 3,3′-diaminobenzidine (DAB).

### 2.10. ELISA Analysis of Inflammatory Cytokines

After treatment, articular cartilage from rats was ground, and the supernatant was collected. Following the instructions of the ELISA kits, the expression levels of inflammatory cytokines—including IL-1β and TNF-α—in cartilage tissues of each group were measured.

### 2.11. Transcriptome Sequencing to Identify Potential Therapeutic Targets

Articular cartilage from rat knee joints was scraped, immediately snap-frozen in liquid nitrogen. cDNA library preparation and sequencing reactions were performed. The quality of the raw data was evaluated by FastQC. The pre-processing of data included background correction, normalization, and mapping valid expression with HISAT2.

### 2.12. Quantitative Real-Time Polymerase Chain Reaction (qRT-PCR) Analysis

The total RNA was extracted from the cells using TRIzol reagent. cDNA was synthesized using a cDNA Synthesis Kit (Roche, Shanghai, China). qRTPCR was conducted to amplify the cDNA using a 7500 Real-time detection system (Applied Biosystems, Waltham, MA, USA). The primers used in this work were designed by Sangon Biotech Co., Ltd. (Shanghai, China), and the sequences of these primers are listed in Table 1.

### 2.13. Statistical Analysis

Data are presented as mean ± standard deviation (mean ± S.D.). Differences between two groups were statistically analyzed by unpaired, two-tailed Student’s *t* test, while analysis of variance (ANOVA) with Dunnett post hoc test was used for the comparison of data in more than two groups of variables. The levels of significance were set at * *p* < 0.05, ** *p* < 0.01, *** *p* < 0.001, and **** *p* < 0.0001; # *p* < 0.05, ## *p* < 0.01, ### *p* < 0.001, and #### *p* < 0.0001, respectively. All statistical analyses were performed with GraphPad Prism software version 8.0 (GraphPad Software, Inc., La Jolla, CA, USA).

## 3. Results and Discussion

### 3.1. Purification and Identification of hUC-MSC-Derived Exosomes

Previous studies have shown that human umbilical cord mesenchymal stem cells have therapeutic potential for OA, and recent evidence suggests that the beneficial effects of MSCs are mainly mediated by secreted exosomes. MSC-derived exosomes can promote tissue repair and reduce inflammation, which has gradually attracted wide attention in the treatment of OA. Although MSC-EXO is a promising alternative for MSC-based therapies for the treatment of OA, large-scale production and purification of MSC-EXO remains a major challenge. In this study, we developed an efficient, rapid, and scalable method for the isolation and purification of MSC-EXOs, as illustrated in Figure 1A. First, hUC-MSCs were cultured in serum-free medium. During continuous culture, the cells grew adherent and exhibited a spindle-shaped morphology under microscopy. The identity of hUC-MSCs was characterized by flow cytometry for surface staining of hMSCs surface markers. These hUC-MSCs were found to be positive for CD73, CD90, and CD105, and negative for HLA-DR as well as the hematological lineage markers CD19, CD34, CD45, and CD11b (Figure 1B). To obtain hUC-MSC-EXOs, the collected culture supernatant was processed using a combination of tangential flow filtration (TFF) and Capto Core 400 multimodal chromatography. The purified exosomes were then characterized by nanoparticle tracking analysis (NTA), transmission electron microscopy (TEM), and Western blotting. NTA was used to characterize exosomes size distribution and concentration, whereas TEM and Western blotting were used to verify vesicular morphology and EV-associated marker proteins, respectively. NTA showed that the particle concentration of the exosomes was 5.8 × 10^10^ particles/mL, with an average diameter of 112.7 nm (Figure 1C). Transmission electron microscopy revealed that the purified exosomes exhibited a biconcave, double-membrane vesicular morphology, with an intact lipid bilayer and clearly visible cup-shaped structures (Figure 1D). Western blot analysis demonstrated that the exosomes expressed classical marker proteins, including CD63, CD9, CD81, and TSG101, all of which appeared as distinct and specific bands (Figure 1E). GAPDH was included as a loading reference.

### 3.2. Exosomes Derived from hUC-MSCs Markedly Alleviate OA Symptoms

**Figure 2:**
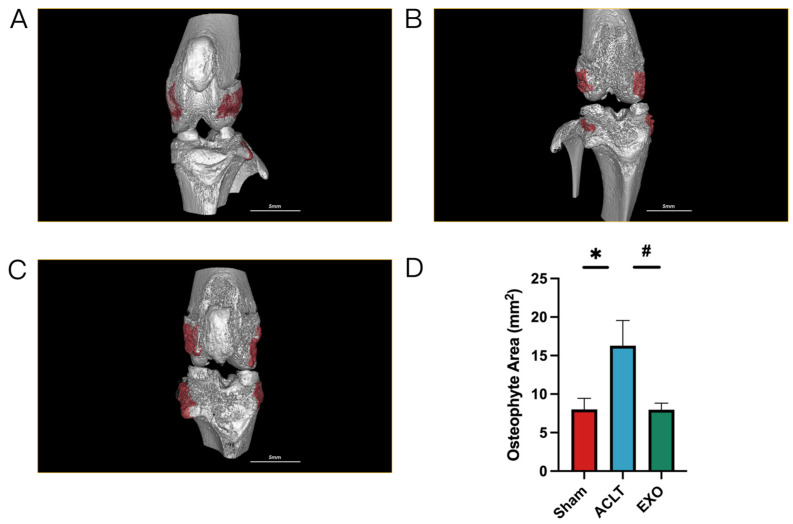
Representative micro-CT images of rat knee joints from the sham control group (**A**), ACLT group (**B**), and exosome-treated group (**C**). Red regions indicate osteophytes (**D**), Figure 2D. Quantification of osteophyte area in reconstructed micro-CT images. Data are presented as mean ± SD (n = 6). * *p* < 0.05 vs. Sham group; # *p* < 0.05 vs. ACLT group.

Compared with the sham-operated control group, rats in the ACLT-induced arthritis group exhibited pronounced osteophyte formation in the knee joint. In contrast, osteophyte formation was significantly reduced in the exosome-treated group compared with ACLT group, indicating that exosomes can ameliorate OA-related osteophyte development (Figure 2).

**Figure 3:**
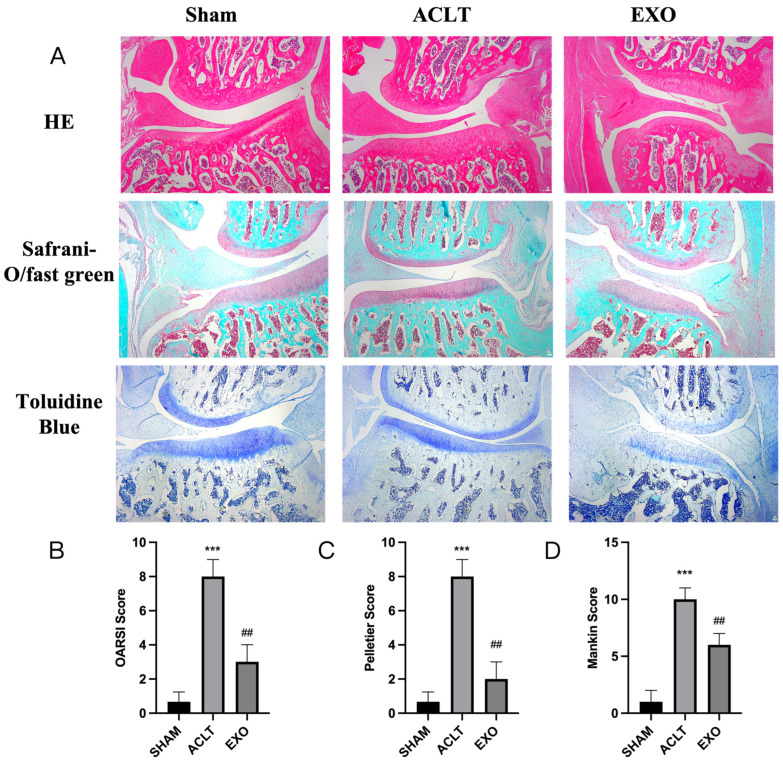
Representative images of HE staining, Safrani-O/fast green staining, and toluidine blue staining of knee joint sections of OA model rats with various intervention. Magnification: 40×. (**A**) Representative images of toluidine blue staining of knee joint sections of OA model mice with various intervention. Scale bar = 100 μm. Summary results of OARSI score (**B**), Pelletier Score (**C**), and Mankin Score (**D**) knee joint sections of OA model mice with various intervention. Data are presented as mean ± SD. n = 6, *** *p* < 0.001 vs. Sham group; ## *p* < 0.01 vs. ACLT group.

Histopathological analysis revealed that the cartilage of the sham-operated control group showed an intact architecture, a smooth articular surface, uniform matrix staining, and no apparent extracellular matrix damage or inflammatory cell infiltration. In the ACLT group (OA model group), severe cartilage degeneration was observed, including a roughened surface, diminished matrix staining, exposure of the subchondral bone, and disorganized chondrocyte arrangement, along with marked synovial inflammation, which features indicative of advanced degenerative joint lesions. Compared with the ACLT group, the exosome-treated group (EXO) showed reduced cartilage structural destruction, improved matrix staining, more orderly chondrocyte arrangement, and markedly attenuated synovial inflammation, suggesting that these exosomes exert potent protective effects on cartilage structure and inflammatory response (Figure 3A).

Quantitative scoring of histological staining further demonstrated that the OARSI score of the ACLT model group was significantly higher than that of the normal control group, indicating severe cartilage degeneration. Similarly, the synovitis score and cartilage fissure score in the ACLT group were significantly elevated compared with the controls, reflecting substantial synovial inflammation and pronounced cartilage surface damage. After exosome treatment, all scores showed varying degrees of reduction, indicating that exosomes exert a mitigating effect on OA-associated pathological changes (Figure 3B–D).

**Figure 4:**
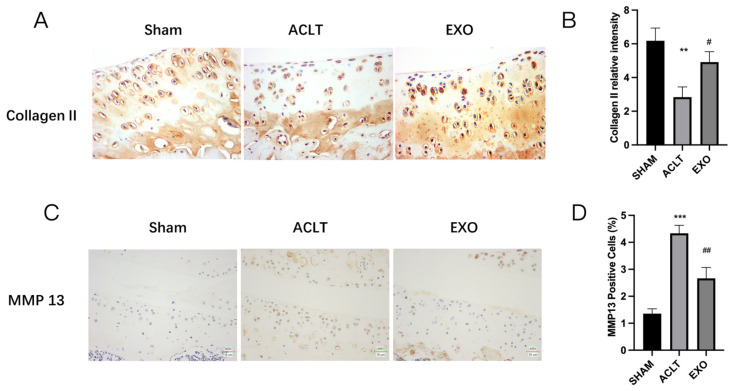
Exosome treatment markedly promoted Collagen II expression and suppressed MMP13 expression during ACLT-induced cartilage degeneration. (**A**) Representative immunohistochemical images of Collagen II staining. (**B**) Quantitative analysis of relative Collagen II expression. (**C**) Representative immunohistochemical images of MMP13 staining. Magnification: 400×, scale bar: 10 μm. (**D**) Quantitative analysis of the proportion of MMP13-positive cells. Data are presented as mean ± SD. n = 6. ** *p* < 0.01, *** *p* < 0.001 vs. Sham group; # *p* < 0.05, ## *p* < 0.01 vs. ACLT group.

To evaluate the protective effects of exosomes (EXO) on cartilage matrix components, immunohistochemistry was performed to examine the expression of type II collagen (Collagen II) in the articular cartilage of rat knee joints across different groups. As shown in the figure, both the superficial and deep zones of the cartilage in the Sham group displayed strong, brownish-yellow positive staining for Collagen II, indicating abundant expression and intact cartilage architecture (Figure 4A). In contrast, the ACLT group showed markedly attenuated staining, suggesting substantial loss of type II collagen and severe degradation of the cartilage matrix. Compared with the ACLT group, the Exo-treated group exhibited markedly enhanced staining intensity, indicating that exosome intervention effectively attenuated ACLT-induced cartilage matrix degradation. Quantitative analysis revealed that Collagen II expression was significantly reduced in the ACLT group compared with the Sham group, whereas its expression was markedly higher in the EXO group than in the ACLT group, further confirming the protective role of exosomes in maintaining cartilage integrity (Figure 4B).

To further investigate the role of exosomes (EXO) in suppressing cartilage matrix degradation, the expression of matrix metalloproteinase-13 (MMP13) in articular cartilage was detected. As shown in Figure 4C, only a few MMP13-positive cells were observed in the Sham group, with minimal brown staining signals, indicating baseline physiological levels. However, MMP13 expression was markedly increased in the ACLT group, with numerous chondrocytes exhibiting positive staining, suggesting significantly elevated cartilage-degrading activity. Following EXO treatment, the number of MMP13-positive cells was notably reduced, and staining intensity weakened, indicating that exosomes effectively suppress the expression of matrix-degrading enzymes. Quantitative results (Figure 4D) showed that the proportion of MMP13-positive cells in the ACLT group was significantly higher than in the Sham group, whereas the EXO group showed a pronounced reduction compared with the ACLT group, suggesting that exosomes effectively inhibit inflammation-related enzyme expression during OA progression and exert protective effects on the cartilage matrix.

**Figure 5:**
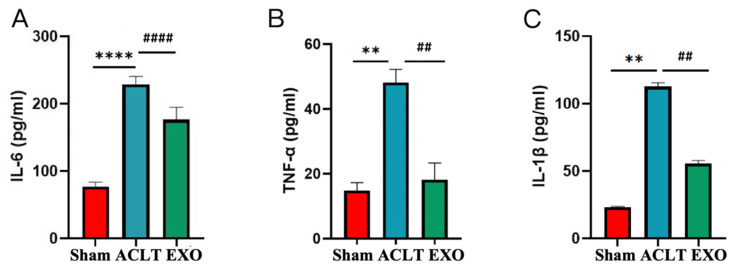
Effects of exosome (EXO) treatment on the expression of inflammatory cytokines in ACLT-induced osteoarthritis. Expression levels of IL-6 (**A**) and TNF-α (**B**), as well as IL-1β (**C**), in knee joint tissues from different groups. Data are presented as mean ± SD. ** *p* < 0.01, **** *p* < 0.0001 vs. Sham group; ## *p* < 0.01, #### *p* < 0.0001 vs. ACLT group.

To evaluate the regulatory effects of exosome treatment on inflammatory responses, the expression levels of inflammation-related cytokines, including IL-6, TNF-α, and IL-1β, were examined in joint tissues. The results showed that measured inflammatory cytokines were markedly upregulated in the ACLT group, whereas their levels were significantly reduced following exosome treatment (Figure 5), indicating that exosomes effectively ameliorate the local inflammatory microenvironment associated with OA [38].

### 3.3. Transcriptomic Analysis of Potential Therapeutic Targets Associated with hUCB-MSC-Derived Exosome Treatment for Osteoarthritis

Based on the results described above, exosomes derived from hUCB-MSCs significantly alleviated osteoarthritis symptoms. To further elucidate their underlying mechanisms, articular cartilage samples from each group of rats were collected for transcriptome sequencing, and potential therapeutic targets and regulated signaling pathways were identified through bioinformatic analysis.

**Figure 6:**
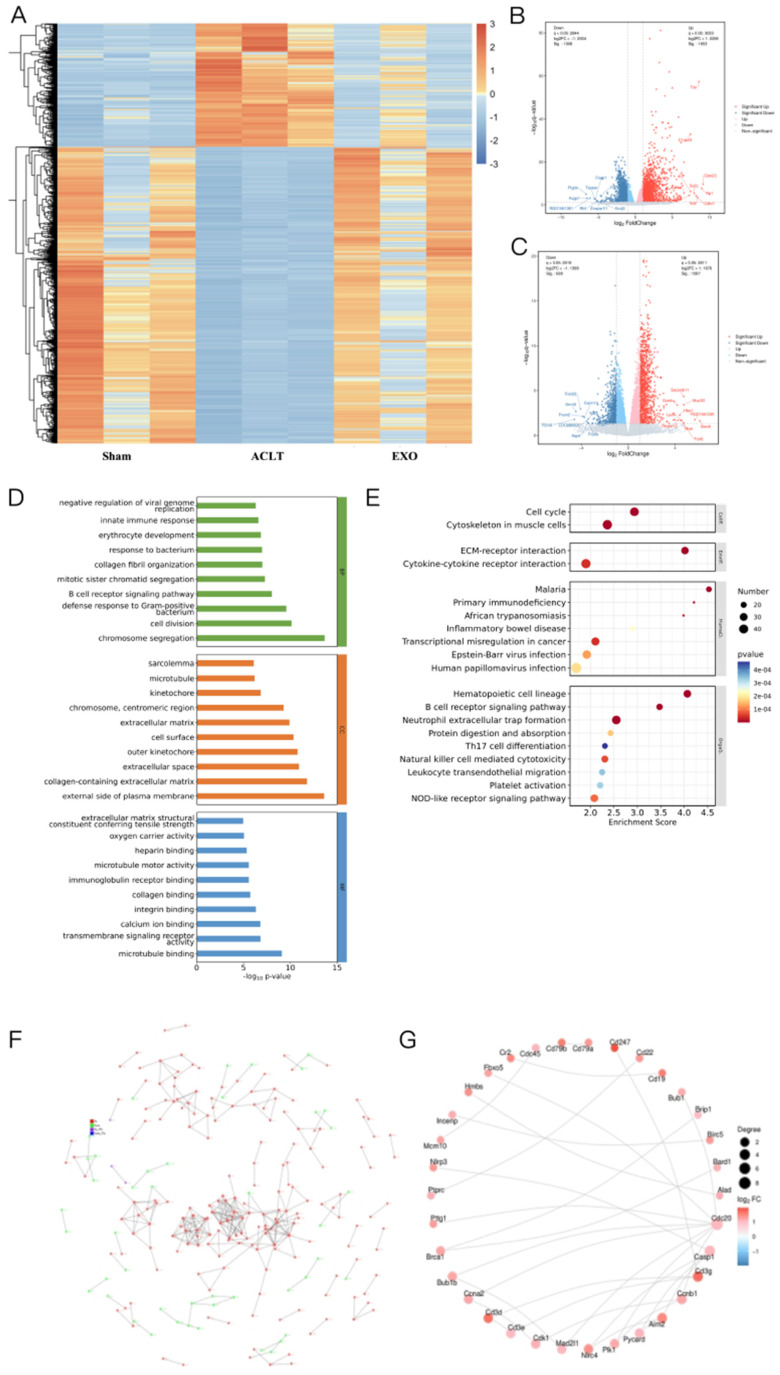
Integrated transcriptomic analysis of hUCB-MSC-derived exosome treatment in ACLT-induced osteoarthritis. (**A**) Hierarchical clustering heatmap of differentially expressed genes among the sham, ACLT, and EXO groups. (**B**) Volcano plot showing DEGs between the sham and ACLT groups. (**C**) Volcano plot showing DEGs between the ACLT group and the EXO group. Blue dots represent significantly downregulated genes, and red dots represent significantly upregulated genes. (**D**) GO enrichment analysis, and (**E**) KEGG enrichment bubble plot comparing the EXO group with the ACLT group. The *x*-axis represents the enrichment score; larger bubbles indicate pathways with a greater number of enriched protein-coding DEGs, and bubble colors ranging from blue to white to yellow to red correspond to decreasing *p*-values and increasing significance. (**F**) Protein–protein interaction (PPI) of the top 300 differentially expressed genes between the ACLT group and the EXO group. (**G**) PPI network of the top 30 DEGs between the ACLT group and the EXO group.

A total of 5437 expressed genes were detected. Hierarchical clustering of the high-throughput sequencing results and differentially expressed genes (DEGs) from the sham control group (Sham), ACLT induce OA group (ACLT), and exosome-treated group (EXO) reflected gene expression correlations both within and between groups. The DEG clustering heatmap is shown in Figure 6A, where red indicates relatively high-expression protein-coding genes and blue indicates relatively low-expression protein-coding genes.

Comparison of DEGs between the Sham group and the ACLT group identified 1653 significantly upregulated genes, with the top ten including *Tnn*, *C1qtnf3*, *Cldn23*, *Rp1*, *Cdnr1*, *Sctr*, *Cfi*, *Sv2c*, *Cdhr4*, and *Tvrp*. A total of 1308 genes were significantly downregulated, with the top ten including *Azpg1*, *Ptgds*, *Tspear*, *Csap1*, *RGD1561381*, *Rtl4*, *Zcwpw1l1*, and *Kcnj3*. Comparison of DEGs between the EXO group and the ACLT revealed 1097 significantly upregulated genes, with the top ten including *Serpinb11*, *RGD15621381*, *Muc3l1*, *Gzmb*, *Lyzl4*, *Hbe1*, *Rnase12*, *Grpr*, *Grm8*, and *Fcrl65*. A total of 639 genes were significantly downregulated, with the top ten including *Trim29*, *Dmrt2*, *Garin1b*, *Frem2*, *Tp63*, *P2rx6*, *LOC689303*, *Aqp4*, *Pcare*, and *Angph7* (Figure 6B,C).

GO enrichment analysis was performed on the differentially expressed genes (DEGs) identified above to characterize their functional implications based on GO annotation, including biological process (BP), cellular component (CC), and molecular function (MF). The BP analysis revealed that many DEGs were involved in downregulated biological processes such as cell division, chromosome segregation, DNA replication, and DNA repair. In contrast, DEGs were upregulated in processes related to the positive regulation of cell migration, extracellular matrix organization, negative regulation of cell chemotaxis, cell adhesion, and activation of the classical complement pathway. In the CC category, DEGs were downregulated in terms associated with the extracellular matrix, collagen trimer, collagen-containing extracellular matrix, and astrocyte-related components, whereas they were upregulated in pathways involving the extracellular space, extracellular matrix, collagen-containing extracellular matrix, and the external side of the plasma membrane. MF analysis demonstrated that DEGs were significantly upregulated in nucleic acid binding, sodium ion binding, structural constituents conferring tensile strength, and BMP binding. Conversely, downregulated functions included growth factor activity, extracellular matrix binding, proteoglycan binding, calcium ion binding, collagen binding, and receptor-related functions (Figure 6D).

The top 20 KEGG pathways (filtered by PopHits ≥ 5 and ranked by −log10 *p*-value from highest to lowest) are shown in the bubble plot (Figure 6E). Key enriched signaling pathways included the Wnt signaling pathway, TGF-β signaling pathway, ECM–receptor interaction, PI3K–Akt signaling pathway, cell cycle pathway, and cytoskeleton-related pathways in muscle cells. In the PPI network, both Cd247 and Cd3d were significantly upregulated (Figure 6F,G). Significant pathways and key genes associated with exosome-based stem cell therapy for osteoarthritis included ECM–receptor interaction, cytokine–cytokine receptor interaction, PI3K–Akt signaling pathway, TGF-β signaling pathway, and Wnt signaling pathway (Table 2). These identified candidate genes warrant further investigation in future studies.

### 3.4. qPCR Validation Partially Supports Transcriptomic Findings

**Figure 7:**
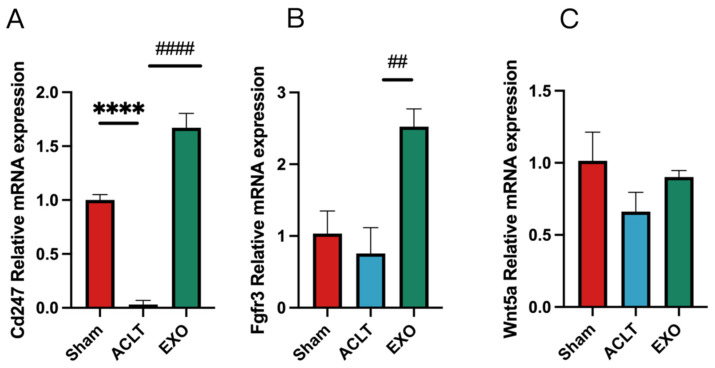
qPCR validation of representative candidate genes associated with exosome-mediated protection in osteoarthritis. Relative mRNA expression levels of *Cd247* (**A**), *Fgfr3* (**B**), and *Wnt5a* (**C**) in the Sham, ACLT, and EXO groups. Data are presented as mean ± SD. N = 6, **** *p* < 0.0001 vs. Sham group; ## *p* < 0.01, #### *p* < 0.0001 vs. ACLT group.

To validate the RNA-sequencing results, qPCR was performed for three representative candidate genes, including *Cd247*, *Fgfr3*, and *Wnt5a* (Figure 7). Compared with the Sham group, *Cd247* expression was markedly reduced in the ACLT group and significantly restored following exosome treatment, indicating that exosomes may regulate immune-related signaling pathways during osteoarthritis progression. In contrast, *Fgfr3* expression was significantly increased in the EXO group compared with the ACLT group, suggesting a potential role in cartilage remodeling or reparative processes associated with exosome-mediated protection. Additionally, *Wnt5a* exhibited a trend toward normalization after exosome treatment, which was generally consistent with the transcriptomic prediction of Wnt signaling pathway involvement. Taken together, these results partially validated the transcriptomic data and further suggest that hUCB-MSC-derived exosomes exert therapeutic effects through coordinated regulation of immune response and cartilage-related signaling pathways.

## 4. Discussion

In the present study, we applied a strategy combining tangential flow filtration and Capto Core 400 multimodal chromatography for the purification of hUC-MSC-derived extracellular vesicles. Using an ACLT-induced OA model, we demonstrated that purified exosomes significantly attenuated cartilage degeneration, reduced inflammatory cytokine production, inhibited osteophyte formation, and improved histopathological outcomes. Transcriptomic and qPCR analyses further suggested that these protective effects may involve coordinated regulation of immune responses, cartilage homeostasis, and tissue repair pathways.

Extracellular vesicles derived from mesenchymal stem cells have attracted increasing attention as a promising cell-free therapeutic approach for osteoarthritis. Compared with conventional ultracentrifugation-based methods, TFF provides improved scalability and reduced processing time, while Capto Core 400 chromatography facilitates the efficient removal of soluble protein contaminants and other impurities [39]. These characteristics may enhance batch consistency and support future large-scale manufacturing and translational applications of MSC-derived exosomes.

Micro-CT and histological analyses consistently demonstrated the therapeutic benefits of hUC-MSC-derived exosomes in OA. Osteophyte formation is a characteristic feature of OA progression and reflects abnormal joint remodeling during chronic degeneration [1]. In the present study, ACLT-induced OA resulted in marked osteophyte formation and structural deterioration of the knee joint, whereas exosome treatment significantly reduced osteophyte burden. Histological evaluation further revealed preservation of cartilage architecture and reduced extracellular matrix loss following exosome administration. These observations were supported by immunohistochemical findings showing increased collagen II expression and decreased MMP13 expression in exosome-treated animals. Collagen II is a major structural component of articular cartilage, whereas MMP13 is a critical matrix-degrading enzyme implicated in cartilage destruction [40]. Together, these findings suggest that hUC-MSC-derived exosomes effectively preserve cartilage integrity and suppress pathological joint remodeling during OA progression.

Inflammation is increasingly recognized as a central driver of osteoarthritis progression and contributes to cartilage degradation, synovial dysfunction, and abnormal joint remodeling [38]. In the present study, exosome treatment significantly reduced the levels of both IL-1β and TNF-α in joint tissues, indicating a substantial attenuation of the inflammatory microenvironment. The observed reduction in inflammatory cytokines may therefore represent an important mechanism by which hUC-MSC-derived exosomes protect cartilage integrity and slow OA progression.

Based on RNA-sequence analysis, among these differentially expressed genes, several have previously been associated with biological processes relevant to osteoarthritis [38,40,41,42,43,44]. For example, C1qtnf3 has been implicated in cartilage homeostasis and inflammatory regulation, whereas Cfi participates in complement-mediated immune responses. Ptgds is involved in prostaglandin metabolism and has been linked to osteoarthritis progression and joint inflammation. Following exosomes treatment, genes such as Gzmb, Grpr, and Tp63 were differentially regulated. Gzmb is associated with immune regulation and inflammatory responses, Grpr has been implicated in tissue repair and cellular signaling, while Tp63 plays important roles in epithelial differentiation and tissue remodeling. Collectively, these findings suggest that exosomes treatment may influence multiple biological processes related to inflammation, immune regulation, and tissue repair in osteoarthritis. The top 20 KEGG pathways are associated with maintaining cartilage homeostasis and regulating bone remodeling, suggesting a potential structural and mechanical regulatory role in OA progression [41,42,43]. Furthermore, pathways such as hematopoietic cell lineage, cytokine–cytokine receptor interaction, and B cell receptor signaling may mediate immune responses following inflammation and play critical roles in immune regulation and tissue repair [43,44]. In the PPI network, both Cd247 and Cd3d were significantly upregulated (Figure 6F,G). Cd247 encodes the CD3ζ protein, which is primarily expressed in NK cells and T cells and functions as part of the signal amplification pathway in T lymphocytes and NK cells. CD247 is closely associated with inflammation, and studies have shown that restoring CD3ζ expression on the surface of T lymphocytes may help reduce tissue inflammation [45,46]. Fgfr3 showed differential expression in transcriptomic analysis, and its upregulation after exosome treatment was further confirmed by qPCR. Fgfr3 encodes the FGFR3 protein, which is expressed in chondrocytes and mature osteoblasts and plays a key role in regulating bone growth. Overactivation of FGFR3 signaling severely suppresses the proliferation and maturation of growth plate chondrocytes [47].

Several limitations should be acknowledged. First, although transcriptomic and qPCR analyses identified several candidate pathways and genes, mechanistic validation was not performed. Second, long-term safety and biodistribution of exosomes were not evaluated. Finally, the therapeutic efficacy was assessed in a single animal model, and future studies are required to confirm these findings in additional preclinical settings.

## 5. Conclusions

In this study, we developed a scalable purification method for hUC-MSC-Exos based on tangential flow filtration (TFF) combined with Capto Core 400 multimodal chromatography, and evaluated the therapeutic effects in osteoarthritis. The purified exosomes exhibited typical characteristics, high concentration, and effectively alleviated ACLT-induced osteoarthritis by reducing cartilage degeneration, suppressing osteophyte formation, preserving collagen II, and inhibiting MMP13 expression. Exosome treatment also mitigated joint inflammation through downregulation of IL-1β and TNF-α. Transcriptomic analysis identified multiple exosome-regulated pathways, including ECM–receptor interaction, PI3K–Akt, TGF-β, and Wnt signaling, which may underlie their chondroprotective effects. qPCR validation further supported these findings, with *Cd247* restored, *Fgfr3* upregulated, and *Wnt5a* showing a trend toward normalization. Overall, these findings highlight the therapeutic potential of hUC-MSC exosomes as a promising cell-free strategy for osteoarthritis management.

## Figures and Tables

**Table 1:** Table of primers used for quantitative PCR.

Gene	Primer Direction	Primer Sequence
*Cd247*	Forward	AGTCCTCGCCTGCATCCTTCAA
	Reverse	GGGTTCCTCCTCCTCTGCTGTTT
*Fgfr3*	Forward	CGGCTACCTGTGAAGTGGATGG
	Reverse	TGTGCAGTTGGCTGGCTTGT
*Wnt5a*	Forward	GTGATGCAAATAGGCAGCCG
	Reverse	ATAGTCGATGTTGTCCCCGC
*Gapdh*	Forward	AGCCCAGAACATCATCCCTG
	Reverse	CACCACCTTCTTGATGTCATC

**Table 2:** Signaling pathways and key genes associated with hUCB-MSC-derived exosome therapy for osteoarthritis.

id	Term	Gene ID
rno04512	ECM-receptor interaction	*Cd36*; *Col4a2*; *Col6a1*; *Col6a2*; *Col6a3*; *Col6a4*; *Comp*; *Frem1*; *Gp1ba*; *Gp5*; *Gp6*; *Gp9*; *Hspg2*; *Itga11*; *Itga2b*; *Itgb7*; *Lama2*; *Lama4*; *Lamc2*; *Sv2c*; *Thbs2*; *Thbs3*; *Thbs4*; *Tnc*; *Vtn*
rno04060	Cytokine–cytokine receptor interaction	*Bmp15*; *Ccl21*; *Ccr1l1*; *Ccr2*; *Cntfr*; *Csf3r*; *Cxcr2*; *Eda2r*; *Epor*; *Gdf5*; *Il11ra1*; *Il12a*; *Il12rb2*; *Il17ra*; *Il17rc*; *l18rap*; *Il21r*; *Il27ra*; *Inhba*; *Ltb*; *Mpl*; *Pf4*; *Ppbp*; *Relt*; *Tnfrsf12a*; *Tnfrsf13b*; *Tnfrsf14*; *Tnfsf14*
rno04151	PI3K-Akt signaling pathway	*Brca1*; *Ccne1*; *Ccne2*; *Cd19*; *Col4a2*; *Col6a1*; *Col6a2*; *Col6a3*; *Col6a4*; *Comp*; *Csf3r*; *Epor*; *Itga11*; *Itga2b*; *Itgb7*; *Lama2*; *Lama4*; *Lamc2*; *Myb*; *Nr4a1*; *Ntrk2*; *Pik3cd*; *Ppp2r3a*; *Syk*; *Thbs2*; *Thbs3*; *Thbs4*; *Tnc*; *Vtn*
rno04350	TGF-beta signaling pathway	*Chrd*; *Fbn1*; *Gdf5*; *Grem2*; *Inhba*; *Nbl1*
rno04310	Wnt signaling pathway	*Fzd1*; *Fzd2*; *Fzd7*; *Fzd8*; *Nfatc4*; *Rspo2*; *Wnt5a*

## Data Availability

Data will be made available upon request.

## Abbreviations

The following abbreviations are used in this manuscript:

OA	Osteoarthritis
MSCs	Mesenchymal stem cells
MSC-EXOs	Mesenchymal stem cell-derived exosomes
hUC-MSC-EXOs	human umbilical cord mesenchymal stem cells-derived exosomes
BMSCs	bone marrow mesenchymal stem cells
SMSCs	Synovial mesenchymal stem cells
AMSCs	adipose-derived mesenchymal stem cells
TFF	tangential flow filtration
ACLT	anterior cruciate ligament transection
NTA	nanoparticle tracking analysis
TMP	transmembrane pressure
TEM	Transmission electron microscopy
ROI	region of interest
OARSI	Osteoarthritis Research Society International
H&E	hematoxylin and eosin staining
DAB	3,3′-diaminobenzidine
Collagen II	type II collagen
MMP13	matrix metalloproteinase-13
DEGs	differentially expressed genes
PPI	Protein–protein interaction
BP	biological process
CC	cellular component
MF	molecular function
